# Proceedings of the Buffalo Medical Association

**Published:** 1877-10

**Authors:** Bernard Bartow

**Affiliations:** Secretary, Pro Tem.


ART. III.—*Abstract of the Proceedings of the Buffalo Medical Association.* Tuesday, evening, September 4th, 1877. Reported by Bernard Bartow, M. D., Secretary, *Pro Tem.*

The meeting was called to order by the vice-president, Dr. Thos. Lothrop. In the absence of the secretary, Dr. Bartow was chosen to occupy that position.

Dr. S. W. Wetmore read a paper, entitled "What is Modern Homœopathy?" Under the head of voluntary communication,

Dr. Thos. F. Rochester, presented an unique pathological specimen with the history of the case. (See Art. II, page 93.)

Dr. White thought it was one of the most interesting pathological specimens he had ever seen. It was without a parallel. Did not know whether he could adopt unreservedly, the theory advanced by Dr. Rochester, as to the manner in which the occlusion was caused. Here was a segment of small intestine of several inches in extent, entirely gone—obliterated, with both of the divided ends sealed up by a delicate film-like tissue. It seemed to him a great wonder that such a state of things could take place. He considered it an anomaly, and was personally under many obligations to Dr. Rochester for bringing such an interesting case to his attention, and thought the society would coincide with him in thanking Dr. R. for the report.

Dr. Howe presented a patient upon whom he had performed an operation for enucleation of the eye.

The patient, E. M., was a woman forty years old, who belonged to a healthy family, and who had always been well up to two years before that time; she began to notice a dark colored spot apparently beneath the conjunctiva of the right eye, at its lower and inner portion. This gradually increased in size, and when first examined, on the third of April, the condition presented was as follows: In the lower and internal portion of the right orbit there was a roundish tumor, measuring one and one-eighth inches in horizontal diameter and five-eighths inch vertically. It was dark brown near the base, black at its apex, not tender to touch, had spongy feel, and was somewhat moveable. The lower lid was pushed down and forwards; the globe, up and outwards. The internal and lower quadrant of the cornea was opaque, and the adjacent portion of the sclerotic inflamed. Intraocular changes were slight and vision, one-fifth of normal. Patient had a cachectic appearance, and was losing flesh rapidly. The melanotic nature of the growth was thus made evident, and its removal together with enucleation of the affected globe was decided upon; with the exception of a profuse hæmorrhage, there was nothing unusual about the operation. The wound did not tend to heal readily. Subsequent inflammation was severe, and on the fifth day signs of the tumor were visible near the former site. The patient was therefore chloroformed again, and the greatest part of the contents of the orbit was removed. The effect of this was more satisfactory. The wound healed readily, and within three weeks was converted into a firm, healthy cicatrix. This appearance it retains until the present time—five months after the operation. The general condition of the patient meanwhile improving much.

The case was a good illustration of many similar ones, in regard to its history and treatment. If left to follow out the usual course, it would, undoubtedly have taken on a more malignant character, and have terminated fatally, nor did a removal of the diseased portion alone suffice to eradicate it. For this reason, some practitioners seeing similar growths return after one such thorough, but unsccessful attempt, are apt to deprecate interference in any form. When, however, the adjacent tissues, though of a healthy appearance, are not spared, then the result is satisfactory.*

A section of the tumor which presented the appearance known as melanotic sarcoma was shown under a demonstrating microscope, and remarks made upon the general characteristics of allied growths.

The president upon motion, appointed a committee to lay out a plan of work for the association, and secure papers for the next meeting. The committee appointed was Drs. Lucien Howe, P. W. Van Peyma and L. F. Harvey.

Dr. Mynter was appointed essayist for the next meeting.

On motion, the association adjourned.

———:o:———

### Correspondence.

UPTONVILLE, Sept. 4th, 1877.

*Editors Medical Journal:*

At the conclusion of my last letter I promised to give you some account of my experience in the care of the pauper population. I now beg to be excused until another time, prefering at this time to detail how some of the medical profession at Uptonville gain

Five months time must be regarded as much too short to determine results. We have known similar disease to remain stationary seven years after removal, yet return to prove fatal at end of eight years. (ED.)

